# Research progress on the application of multi-scale computational methods in the research and development of anti-tuberculosis infection drugs

**DOI:** 10.3389/fphar.2026.1869128

**Published:** 2026-10-14

**Authors:** Yajing Zhao, Chao Wang, Wenjing Shang, Xun Peng, Mingyu Zhang, Chuanxi Zhao

**Affiliations:** Department of Clinical Laboratory, Shandong Cancer Hospital and Institute, Shandong First Medical University and Shandong Academy of Medical Sciences, Jinan, Shandong, China

**Keywords:** machine learning, molecular docking, molecular dynamics simulation, network pharmacology, systems pharmacology, tuberculosis

## Abstract

Tuberculosis (TB), driven by *Mycobacterium tuberculosis* (Mtb), remains a leading cause of mortality from infectious disease worldwide.​ The global dissemination of multidrug-resistant (MDR) and extensively drug-resistant (XDR) strains—coupled with protracted treatment regimens and the persistence of latent infection—has not only compromised first-line chemotherapies but also underscored the stagnation of conventional drug development pipelines. In this review, we systematically synthesize how multi-scale computational strategies are reshaping anti-TB drug discovery and explicitly delineate their hierarchical integration—a cohesive framework that, to our knowledge, has yet to be comprehensively mapped in the existing literature.​ We survey four interconnected computational layers: (i) systems and network pharmacology for macroscopic target and pathway prioritization; (ii) machine learning (ML) for mesoscopic virtual screening, resistance classification, and ADMET prediction; (iii) molecular docking for microscopic elucidation of ligand–target binding modes; and (iv) molecular dynamics (MD) simulations for atomic-level validation of binding poses and assessment of complex stability. Representative studies demonstrate that this workflow has pinpointed pivotal TB vulnerabilities (e.g., MmpL3, InhA, DprE1, ATP synthase, and FtsZ), enabled ML models to predict drug resistance from genomic data with high fidelity, and identified promising candidates such as vazabitide A (predicted binding affinity of −85.8 kcal/mol against Pks13) alongside repurposed agents (e.g., aldoxorubicin, quarfloxin). Critically, these computational predictions are reinforced by orthogonal experimental validation spanning *in vitro* enzymatic and phenotypic assays, *in vivo* infection models, clinical pharmacokinetic profiling, and multi-omics corroboration. We conclude that the top-down pipeline-spanning network pharmacology to ML, docking, and MD-integrated with bottom-up feedback constitutes a closed-loop paradigm that accelerates lead identification while addressing emergent drug resistance.​ Nevertheless, inherent limitations in predictive reliability and cross-layer error propagation necessitate rigorous downstream experimental validation. Looking forward, generative artificial intelligence, self-driving laboratories, and standardized cross-method benchmarking are poised to consolidate this multi-scale pipeline into a robust engine for next-generation anti-TB therapeutics.

## 1 Introduction

TB persists as a critical global public health threat: approximately one-quarter of the world’s population is estimated to be infected with Mtb, with 5%–10% of those harboring latent infections progressing to active disease over their lifetimes (Vasiliu et al., 2024). Each year, an estimated 10 million individuals develop active TB, resulting in 1.4 million deaths (Kaul et al., 2023). In 2022, 10.6 million new cases were reported worldwide, solidifying TB’s position as the second leading cause of death from a single infectious agent. The geographical distribution of incident cases remains highly heterogeneous: over two-thirds occurred in eight high-burden countries—India (26%), Indonesia (10%), China (6.8%), the Philippines (6.8%), Pakistan (6.3%), Nigeria (4.6%), Bangladesh (3.5%), and the Democratic Republic of the Congo (3.1%)—underscoring pronounced regional disparities in disease control. As the disruptions wrought by the COVID-19 pandemic receded, the TB death toll rebounded to 1.25 million in 2023, re-establishing TB as the world’s deadliest infectious disease and starkly exposing the structural fragility of global public health systems (Trajman et al., 2022).

Of greater concern is the escalating prevalence of drug-resistant TB. XDR-TB has extremely limited therapeutic options and an extremely poor prognosis, with a historical treatment success rate of less than 30%. Although bedaquiline- and linezolid-based all-oral short-course regimens have markedly improved treatment outcomes for highly drug-resistant TB, with success rates reaching 89%–92%, pediatric and adult patients with XDR-TB still confront substantial obstacles, including divergent treatment regimens, considerable adverse effects, and suboptimal adherence to follow-up. Worldwide, the overall treatment success rate for XDR-TB remains only 50%–60%, which is still insufficient to meet clinical demand (Conradie et al., 2020; Osman et al., 2019). Furthermore, severe adverse events—including drug-induced liver injury, hepatic and renal failure, myelosuppression, and neurotoxicity-frequently precipitate regimen discontinuation and undermine adherence (Lewis et al., 2024; He et al., 2026; Arbex et al., 2010). Accordingly, sustained investment in the discovery of novel anti-TB agents, vaccines, and host-directed therapies remains imperative to surmounting this persistent global health challenge (Luo et al., 2024).

Despite the precipitous decline in TB mortality following the introduction of first-line agents such as isoniazid and rifampicin, the confluence of drug-resistant strains and HIV co-infection has driven a marked resurgence in incidence (Kumar et al., 2015). Yet, despite hundreds of putative anti-TB candidates reported annually, remarkably few advance to clinical development. Consequently, the World Health Organization maintains that the discovery and development of novel anti-TB therapeutics are indispensable to ending the global TB epidemic (Fernandes et al., 2022).

Four interrelated challenges underscore the urgency of integrated computational strategies. First, the complexity of MDR/XDR phenotypes: resistance mechanisms extend beyond single-point mutations to include efflux pump overexpression and metabolic rewiring, necessitating network-level analyses to capture systemic perturbations (Muteeb et al., 2025). Second, the limitations of current lengthy regimens: prolonged treatment duration increases non-compliance and toxicity risks, demanding ML–driven rapid screening and structure-based binding prediction (Huang et al., 2023). Third, the persistence of latent TB infection: the hypoxic, non-replicating state within granulomas alters drug–target binding kinetics, requiring MD simulations to assess efficacy under physiological conditions (Carnero Canales et al., 2026). Fourth, the stagnation of conventional drug discovery: despite the annual reporting of hundreds of molecules, high attrition rates and prohibitive costs continue to impede clinical translation (Galletti et al., 2022). Computational drug repurposing addresses this bottleneck by leveraging established pharmacokinetic and toxicity profiles, drastically compressing preclinical timelines and offering a cost-effective pathway to identify non-obvious therapeutic candidates. Collectively, these challenges cannot be addressed by conventional reductionist approaches, necessitating a paradigm shift toward multiscale computational strategies that integrate systems-level network perturbations with atomic-level binding determinants. Specifically, this “multi-scale computational strategy” refers to a comprehensive workflow comprising data-driven network analysis (systems/network pharmacology), large-scale virtual screening (ML), and physics-based structural validation (molecular docking and dynamics simulations). Against this backdrop, multiscale computational approaches—including systems pharmacology, network pharmacology, ML, molecular docking, and MD simulations—present distinct advantages over conventional experimental methods (Wan et al., 2025; Liu et al., 2026; Woillard et al., 2026). These techniques enable a comprehensive, multi-level dissection of the interplay among drugs, targets, and disease networks, thereby improving the efficiency and accuracy of drug discovery. Notably, the computational repurposing of approved or clinically evaluated drugs for TB has emerged as a highly promising strategy to expedite anti-TB agent discovery (Zheng et al., 2024). However, while these individual techniques hold recognized potential, a comprehensive synthesis specifically mapping their synergistic integration remains lacking. This review aims to fill this gap by providing a cohesive framework to accelerate anti-TB drug discovery. Acknowledging this gap in concrete terms, existing syntheses tend to treat network pharmacology, machine learning, docking, and MD as isolated topics: network-pharmacology reviews rarely reach atomistic validation, docking and MD studies seldom trace back to systems-level disease mechanisms, and ML screening workflows infrequently close the loop with experimental orthogonal evidence. Crucially, none of the available reviews is purpose-built for tuberculosis—most are generic or disease-agnostic—so the TB-specific challenges of MDR/XDR phenotypes, persistence/latency, and lengthy regimens are left unaddressed. By explicitly mapping how the four layers concatenate into a single anti-TB discovery pipeline and grounding every stage in TB literature, this review supplies the integrative, disease-focused perspective that the field currently lacks.

The methodological architecture of this multiscale strategy is outlined below. At the macroscopic level, network and systems pharmacology leverage databases such as Traditional Chinese Medicine Systems Pharmacology Database (TCMSP), GeneCards, Online Mendelian Inheritance in Man (OMIM), and Therapeutic Target Database (TTD) to identify potential targets of natural product components. Protein–protein interaction (PPI) networks are constructed via STRING or Cytoscape, followed by Kyoto Encyclopedia of Genes Genomes/Gene Ontology (KEGG/GO) annotation using DAVID or Metascape (*p* < 0.05, FDR corrected) to systematically identify key signaling pathways and core targets (e.g., TNF-α, TLR) (Yao et al., 2023).

At the mesoscopic level, ML-based virtual screening is conducted against compound libraries such as ChEMBL, PubChem, or ZINC (typically 10^4^–10^6^ entities). ML models—including Random Forest, Support Vector Machines, and Deep Neural Networks—are trained on molecular descriptors (e.g., ECFP4, MACCS keys) using tools such as RDKit, DeepPurpose, or scikit-learn, aiming to efficiently exclude inactive molecules and improve hit rates and druggability (Alnammi et al., 2023). At the microscopic level, molecular docking employs AutoDock Vina, Glide, or GOLD to dock candidates against crystal structures of key Mtb targets (e.g., InhA, DprE1, ATP synthase), applying binding energy thresholds (typically ≤−7.0 kcal/mol) and RMSD ≤2.0 Å as primary screening criteria to resolve ligand-binding modes (Patel et al., 2025). Atomic-level validation​ is performed using MD simulations (GROMACS or AMBER under AMBER99SB/CHARMM36 force fields for 50–200 ns), evaluating complex stability (RMSD <3 Å, Rg fluctuation <0.2 nm, persistence of key hydrogen bonds and hydrophobic interactions), complemented by Molecular Mechanics-Poisson Boltzmann Surface Area (MM/PBSA) or Molecular Mechanics-Generalized Born Surface Area (MM/GBSA) calculations to confirm binding affinity and eliminate false positives (Guendouzi et al., 2025; Steshin et al., 2024). Ultimately, this multi-scale closed-loop strategy aims to identify lead compounds possessing high target specificity, favorable druggability, and dynamic stability, providing reliable candidates for the development of novel anti-TB drugs capable of effectively addressing the challenge of drug resistance (Figure 1). To assist readers in selecting appropriate computational tools and provide a practical reference for medicinal chemists, the major software packages and databases cited in this review, together with their working mechanisms, primary applications in TB research, respective advantages and limitations, and key supporting references, are summarized in Supplementary Table S1. In parallel, the compounds discussed in this review are categorized into five major classes—natural products, FDA-approved drugs, repurposed drugs, synthetic compounds, and investigational candidates—and their corresponding computational workflows are systematically examined in subsequent sections, as outlined in Table 1.

**FIGURE 1:**
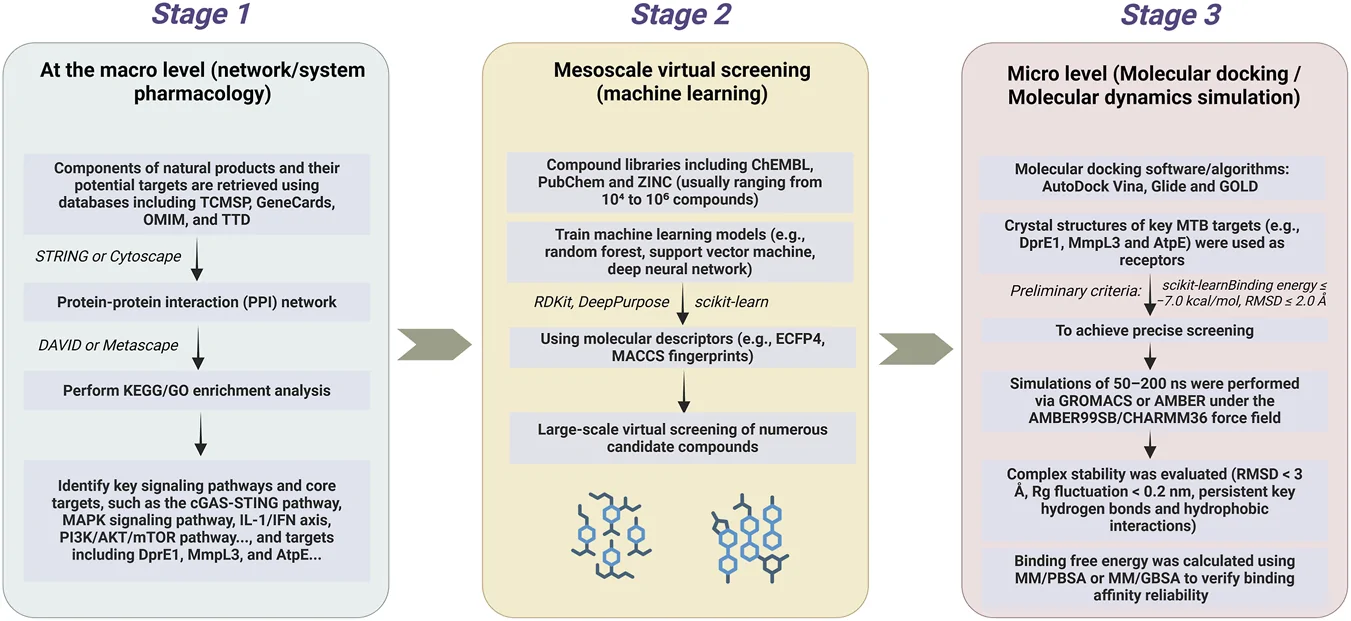
An integrated multi-scale computing framework for anti-TB drug R&D.

**TABLE 1:** Classification of Anti-TB compounds by origin, developmental stage, and computational applicability.

Category	Definition and characteristics	Representative compounds (selected)	Typical computational workflow	Key advantages	Key limitations	Ref.
Natural Products and Derivatives	Plant-, microbe-, or marine-derived scaffolds; often multi-target	*Garcinone D, neodiospyrin, physalin D, fusidic acid, thalirugidine, Acacia nilotica* phytochemicals	Network Pharmacology → Docking → MD	Structural diversity; multi-target synergy; evolutionary optimization	Pre-existing resistance; toxicity at therapeutic doses	(Suresh et al., 2025; Singh and Purohit, 2025; Singh et al., 2024) (Akinpelu et al., 2020; Sharma et al., 2023; Xu et al., 2025)
FDA-Approved Drugs	Marketed drugs with established safety, PK, and formulation	Bedaquiline, isoniazid, rifampicin, pyrazinamide	ML for resistance prediction; Docking/MD for mechanism elucidation	Known safety; existing supply chains; dose–response data	Pre-existing resistance; toxicity at therapeutic doses	(Wu et al., 2020) (Shahbaaz et al., 2022) (Zhang et al., 2020; Luan et al., 2025)
Repurposed Drugs	Approved for other indications; computationally repositioned for TB	Aldoxorubicin, quarfloxin, prazosin, mefloquine	ML virtual screening → Docking → MD validation	Shortened development timeline; reduced preclinical costs; known PK/toxicity	Suboptimal tissue penetration; dose adjustment needs; potential off-target effects	(Yao et al., 2023), (Lee et al., 2024; Dos Santos et al., 2021; Shahab et al., 2024)
Synthetic Small Molecules	Fully synthetic, rationally designed scaffolds	Quinolinone-thiosemicarbazones, spiroquinoxaline-oxadiazoles, 4-amino-1,2,4-triazoles, benzimidazole derivatives, thymidylate kinase inhibitors	*De novo* design → ML scoring → Docking → MD refinement	Tunable physicochemical properties; IP-protectable; scalable synthesis	Higher failure rate; unknown safety; extensive optimization required	(Rawat et al., 2025; Fawibe et al., 2025; Kanchrana et al., 2024; Xia et al., 2025; Valencia et al., 2022) (Venugopala et al., 2020) (Pawar et al., 2025)
Investigational NCEs	Novel entities in preclinical/early clinical development	D060 (PknB inhibitor), NKLF2 mutants, FACS-targeted *de novo* designs, GyrB inhibitors	End-to-end multi-scale integration (network → ML → docking → MD)	High novelty; optimized target engagement; tailored PK/PD	High development cost; uncertain clinical translation; regulatory hurdles	(George et al., 2026; Hanwarinroj et al., 2022; Kashyap et al., 2019; Webster and Shepherd, 2022)

The workflow comprises (Vasiliu et al., 2024): Macro-level​ target identification via systems/network pharmacology (PPI & pathways) (Kaul et al., 2023); Meso-level virtual screening of ChEMBL/PubChem/ZINC libraries using machine learning; and (Trajman et al., 2022) Micro-level validation of drug-target interactions through molecular docking and MD simulations (GROMACS/AMBER), enabling accurate prediction of binding stability and lead optimization.

To contextualize the computational strategies discussed herein, it is instructive to briefly trace the historical evolution of anti-TB drug discovery. The pre-chemotherapy era relied solely on sanatorium care and surgical interventions. The first chemotherapy wave began in 1944 with Selman Waksman’s discovery of streptomycin from soil microorganisms at Rutgers University—the first effective anti-TB drug and a breakthrough that earned him the 1952 Nobel Prize (Webster and Shepherd, 2022; Manu and Rogozea, 2022). This was followed by Jorgen Lehman’s para-aminosalicylic acid (PAS, 1946) and the landmark discovery of isoniazid (1952), independently identified by Squibb and Roche Laboratories, which became a cornerstone of anti-TB therapy (Manu and Rogozea, 2022). The British Medical Research Council’s 1948 randomized controlled trial formally established streptomycin as an effective agent and, more importantly, demonstrated that combination therapy prevents the emergence of drug resistance—a principle that remains foundational to TB treatment today (Waller and Rhee, 2026). Rifampicin, a semisynthetic derivative of rifamycin SV from *Streptomyces mediterranei*, was developed at Lepetit Laboratories in Italy in 1965 and introduced clinically in 1968, enabling treatment duration to be shortened to 9 months (Rajeswaran et al., 2022; Sensi, 1983). However, the decades following the introduction of rifampicin witnessed a long stagnation in TB drug development, coinciding with the resurgence of TB driven by the HIV epidemic and the emergence of MDR/XDR-TB (Shaik and Karpoormath, 2024). This stagnation was reversed only after the publication of the *Mycobacterium tuberculosis* genome in 1998 by the Sanger Institute and Institut Pasteur, which ushered in the modern era of structure-based drug discovery (Sharma et al., 2026). The third wave (2012–present) delivered bedaquiline (Janssen, 2012)—the first new anti-TB drug class in 40 years and the first FDA-approved agent specifically for MDR-TB—followed by delamanid (Otsuka, 2014) and pretomanid (TB Alliance, 2019) (Black and Buchwald, 2021). The subsequent endorsement of the 6-month BPaLM regimen (bedaquiline, pretomanid, linezolid, and moxifloxacin) by the World Health Organization has transformed MDR-TB treatment, with cure rates approaching 90% (Flores et al., 2025). This historical arc reveals a clear evolution in discovery strategies: from empirical soil screening (streptomycin, rifampicin) and serendipitous observation (isoniazid), to rational organic synthesis (PAS, ethambutol), and finally to contemporary structure-based and computational approaches (bedaquiline, pretomanid, delamanid). The multi-scale computational methods reviewed herein thus represent the latest chapter in this trajectory, transforming drug discovery from a largely trial-and-error endeavor into an efficient, data-driven paradigm.

## 2 Multiscale computation-driven anti-TB drug discovery: an integrated strategy from network pharmacology to MD simulation

### 2.1 Macroscopic network mechanism analysis: systems pharmacology and network pharmacology

Systems pharmacology represents an interdisciplinary paradigm that integrates systems biology, pharmacology, omics, and computational modeling to holistically and quantitatively dissect multiscale interaction networks among drugs, targets, signaling pathways, and disease phenotypes. It aims to elucidate complex mechanisms of action, predict efficacy and safety, optimize dosing strategies, and accelerate drug development—particularly for multi-component therapies and rare or complex diseases (Lee et al., 2022; Bai et al., 2023)**.** The conceptual foundations of this discipline were laid by Kitano, who advocated for the use of multiscale data integration to decode the dynamic behaviors of biological systems (Kitano, 2002). Building on this framework, Hopkins formally introduced network pharmacology in *Nature Chemical Biology* (2008), positioning it as a core drug discovery paradigm in which network-based analyses replace linear target-centric approaches, thereby enabling the rational identification of multi-target therapeutics (Hopkins, 2008; Noor et al., 2023). Network pharmacology has demonstrated substantial utility across complex diseases—including cancers, neurodegenerative and cardiovascular disorders, and infectious diseases—and is fundamentally reshaping disease taxonomy, diagnostic strategies, and therapeutic development (Joshi et al., 2025; Nogales et al., 2022). In anti-infective drug discovery, it has proven particularly valuable for deciphering the polypharmacology of traditional Chinese medicine and natural products. For example, integrating network pharmacology with metabolomics and molecular docking revealed that *Castanopsis* honey exerts antibacterial activity against *Streptococcus pyogenes* by inhibiting glutathione peroxidase and tyrosyl-tRNA synthetase, thereby disrupting the bacterial redox network (Yu et al., 2024). Similarly, a component–target–pathway network constructed for the Qingre Jiedu Formula—through integrated transcriptomics and UPLC-Q-TOF-MS analyses—demonstrated its anti-hepatitis B virus activity via modulation of the TGFβ1/Smad4 signaling axis (Jia et al., 2024). Analogous strategies have elucidated the multi-target synergistic mechanisms underlying the activity of *Forsythia suspensa* extract against respiratory syncytial virus (Du et al., 2025)and Tonglong Tea against influenza (Wang et al., 2025).Network pharmacology has demonstrated distinctive value in anti-TB drug discovery and host-directed therapy (HDT). By integrating multi-omics data and computational modeling, recent studies have systematically dissected the molecular mechanisms underlying the anti-TB and immunomodulatory effects of natural product–derived compounds. For instance, one study employing mRNA sequencing and PPI network analysis (STRING database) identified core anti-TB targets—including *AKT1*, *TP53*, *EGF*, *ARF1*, *CD274*, and *PRKCZ*—using topological algorithms (degree, betweenness, and closeness centrality) in Cytoscape. Pathway enrichment further linked these targets to herpes simplex virus one infection, endocytosis, and B cell receptor signaling, providing a robust foundation for target screening and biomarker development. (Yang Y. et al., 2023). Complementary to these immunomodulatory effects, several natural products exert anti-TB activity through the multi-target synergistic regulation of the TNF-α, TLR, and JAK–STAT signaling pathways (Nasare and Bagade, 2025). Notably, network pharmacology has facilitated the rational design of HDTs by mapping multi-target networks for G-protein-coupled receptor (GPCR)-modulating compounds that regulate ferroptosis during Mtb infection, identifying prazosin hydrochloride as an HDT candidate that binds *MAPK3* with high affinity (Luan et al., 2025). In parallel, PPI network analysis of Mtb itself has identified 40 core targets—including FtsZ and Mur—revealing that *Acacia nilotica* phytochemicals disrupt cell division and cell wall biogenesis through direct FtsZ inhibition, thereby exerting potent polypharmacological effects (Suresh et al., 2025). Beyond target identification, network pharmacology provides a quantitative framework for dissecting host–pathogen interactions and guiding HDT development—enabling host targeting, resistance reversal, and optimized combination regimens (Mehta et al., 2022). Integrating network pharmacology with computer-aided drug design (CADD), recent studies have addressed MDR-TB mediated by *pncA* mutations. Systematic analysis of natural compounds against mutant pyrazinamidase (PZase) identified garcinone D and neodiospyrin as promising candidates that restore enzymatic function or stabilize mutant conformations, thereby overcoming PZase-mediated resistance (Singh and Purohit, 2025). Parallel insights into target validation have emerged from studies on MmpL3, a central conduit for mycolic acid transport. Construction of an MmpL3 inhibitor–associated PPI network has elucidated inhibitor dissociation mechanisms and dynamic binding characteristics—including binding stability and egress pathways—providing empirical molecular evidence that reaffirms MmpL3 as a pivotal node for anti-TB drug development (Zhao et al., 2025). Complementary to these efforts, a study targeting bedaquiline (BQ)-resistant *atpE* D32 mutations identified thalirugidine, an alkaloid derived from *Thalictrum foliolosum*, as a potent candidate that circumvents BQ resistance by optimizing electrostatic complementarity, thereby disrupting mycobacterial energy metabolism (Singh et al., 2024). Beyond bacterial targets, network pharmacology has delineated pivotal host signaling cascades that govern defense and immunopathology. These include the cGAS–STING pathway (intricately linked to cytosolic DNA sensing) (Han et al., 2025), the MAPK signaling pathway​ (encompassing the p38, ERK, and JNK sub-pathways, which orchestrate anti-mycobacterial immunity) (Diao et al., 2026), the IL-1/Type I interferon (IFN) axis (Bobba et al., 2024), and the PI3K/AKT/mTOR pathway (Raien et al., 2023). Concurrently, multiple studies have validated key Mtb targets, such as DprE1​ and ATP synthase (*AtpE*) (North et al., 2023; Zhang et al., 2024), which underscore the vulnerability of bacterial cell wall biosynthesis. These studies collectively demonstrate that network pharmacology provides core theoretical support for combating drug-resistant TB and developing novel anti-TB strategies by constructing multidimensional component-target-pathway networks. Collectively, these findings bridge interaction networks, signaling pathways, and target genes, offering a robust paradigm for anti-TB drug discovery. A summary of these representative Mtb targets, their Targets/Signaling Pathways and Therapeutic Applications is provided in Table 2.

**TABLE 2:** Summary of systems pharmacology/network pharmacology applications in Anti-TB drug discovery.

Main application directions	Typical research contents/Cases	Targets/Signaling pathways	Candidate drugs/Clinical application	References (Ref.)
Macroscopic Mechanism Elucidation and Multi-Target Synergy Identification	Employs network pharmacology integrating multi-omics and biological network analysis, utilizing PPI networks and topological algorithms to identify core anti-TB targets and Signaling Pathways	AKT1, TP53, EGF, ARF1, CD274, and PRKCZ, Herpes simplex virus 1 infection; Endocytosis; B cell receptor signaling pathway	providing a robust theoretical foundation and molecular insights for drug target screening, mechanistic elucidation, and novel diagnostic biomarker development	(Mehta et al., 2022)
	To reveal the anti-TB mechanism of natural products via multi-target synergistic inhibition of the TNF-α, TLR, and JAK-STAT signaling pathways	TNF-α, TLR, and JAK-STAT signaling pathways	For the identification of anti-TB bioactive natural compounds, such as alkaloids, flavonoids, terpenes, and polyphenols	(Suresh et al., 2025)
	To construct a GPCR compound-regulated ferroptosis-TB “multi-target multi-pathway” network and reveal the HDT mechanism of prazosin against TB by targeting MAPK3	GPCRs, MAPK3, ferroptosis	GPCR-targeting compounds, particularly prazosin hydrochloride, represent promising candidates for the development of host-directed therapies against TB.	(Lee et al., 2024)
	Construct a PPI network of Mtb and reveal the anti-TB mechanism of active ingredients exerting multi-target synergistic inhibition by targeting FtsZ and other proteins	Mtb FtsZ	The selected candidate phytochemicals—chlorogenic acid and ellagic acid—exhibit potent anti-mycobacterial activity	(Singh and Purohit, 2025)
	Screened and characterized the regulatory effects of the natural compounds garcinone D and neodiospyrin on mutant pyrazinamidase (PZase, encoded by the pncAgene)​ targets	Target: Pyrazinamidase (PZase) Bioactive Compounds: Garcinone D, Neodiospyrin	Natural compounds garcinone D and neodiospyrin target mutant PZase, eliciting anti-TB effects through enzyme stabilization and activity restoration	(Singh et al., 2024)
	Study elucidated the dissociation mechanisms and dynamic characteristics of MmpL3 inhibitors, through the construction of an MmpL3 inhibitor-associated PPI network	MmpL3	Study elucidated the dissociation mechanisms and dynamic characteristics of MmpL3 inhibitors, reaffirms MmpL3​ as a pivotal target for anti-TB drug development	(Han et al., 2025)
	Research targeting BQ-resistant *atpE* subunit-c mutations (D32 locus)​ identified thalirugidine​ as the most potent alkaloid derived from *Thalictrum foliolosum*. Thalirugidine overcame the binding deficiency induced by these mutations through the optimization of electrostatic complementarity	Thalirugidine	Thalirugidine exerts anti-TB effects by disrupting bacterial energy metabolism​ and stands as a promising candidate to circumvent BQ resistance	(Akinpelu et al., 2020)
HDT Network Construction	Systematically dissect the interaction network to provide quantitative decision support for host-targeted, resistance-overcoming, and combination-optimized HDT	Inhibitors of mTORC1, HMG-CoA reductase, HDAC and AKT	HDTs targeting host–pathogen interactions represent innovative strategies against Mtb infection	(Zhao et al., 2025)

Key mechanisms of HDTs, targeting host–pathogen interactions include autophagy induction, modulation of host epigenetics, and regulation of cytokine- and lymphocyte-mediated responses.

### 2.2 Mesoscopic data-driven intelligent accelerated screening: application of ML in anti-TB research

Although systems and network pharmacology elucidate disease–target interactions at the macroscopic level, they often yield an unwieldy proliferation of candidate targets and bioactive components, rendering exhaustive experimental validation intractable. Conventional computational pipelines further encounter efficiency bottlenecks when navigating the vast chemical space and interpreting complex genomic determinants of drug resistance. To address these challenges, ML has emerged as a core, data-driven engine capable of prioritizing network-derived hypotheses and performing rapid dimensionality reduction on large-scale compound libraries. This integration not only streamlines the transition from systems-level insights to tractable hit lists but also facilitates seamless progression to downstream atomistic simulations, thereby bridging the gap between network pharmacology and structure-based drug discovery.

In the field of anti-TB drug development, ML has been deeply integrated throughout the entire lifecycle ranging from target identification and hit compound screening to precise clinical therapy. Against the severe challenge of drug-resistant tuberculosis (DR-TB), ML strategies accelerate novel drug discovery and diagnostic-therapeutic optimization via multidimensional approaches. According to data scale, task type and interpretability requirements, machine learning strategies applied in anti-tuberculosis drug discovery and drug resistance inference can be mainly classified into representative paradigms such as traditional shallow models, deep learning approaches, and ensemble and consensus modeling. Traditional shallow models, such as support vector machines (SVM), random forests (RF), and gradient boosting classifiers (GBC), are widely used for anti-TB compound activity classification, ADMET prediction, and resistance inference from whole-genome sequencing data, owing to their robustness on medium-sized datasets and relatively high transparency (Xu et al., 2025; Shahab et al., 2024; Yang et al., 2018; Kouchaki et al., 2019); Deep learning approaches, including deep neural networks (DNN) and graph neural networks (GNN), excel at extracting hierarchical features from large-scale chemical libraries and have become increasingly important in the identification of novel anti-tuberculosis hits (Niazi, 2025; Lane et al., 2018); Bayesian modeling offers a probabilistic framework well suited to early-stage anti-tuberculosis drug screening with limited experimental data (Ekins et al., 2017). Ensemble and consensus modeling, which integrate multiple algorithms or molecular representations, further improve predictive stability in anti-tuberculosis drug discovery and resistance inference. Increasingly, explainable ML (e.g., SHAP analysis) is being adopted to quantify the contribution of key anti-tuberculosis drug resistance mutations or molecular substructures of candidate agents, thereby enhancing biological credibility (Xu et al., 2025; Yang et al., 2018). However, the robustness of these models heavily depends on the quality of training data; dataset bias arising from limited chemical diversity of anti-tuberculosis compound libraries or geographic skew in clinical *M. tuberculosis* isolates can lead to overfitting and poor generalization. Rigorous validation strategies, such as nested cross-validation and external prospective testing, are therefore essential to ensure translational reliability in anti-tuberculosis drug development beyond retrospective benchmarks.

ML has demonstrated exceptional utility in virtual screening, hit identification, and drug repurposing. By integrating ML with deep learning architectures, researchers screened the DrugBank database and experimentally validated two novel anti-TB candidates-aldoxorubicin and quarfloxin-that target Mtb DNA gyrase (Zheng et al., 2024). Against the specific target thymidylate kinase (TMK), support vector machines (SVM) identified three high-affinity inhibitors from large-scale compound libraries (Shahab et al., 2024). Expanding this paradigm, an ensemble of 103 classification models—generated by integrating six molecular representations with 7 ML algorithms and trained on large-scale ChEMBL data—enabled efficient activity prediction across five Mtb targets (Rawat et al., 2025). Beyond screening, ML-driven *de novo* drug design (DNDD) targeting fatty acyl-CoA synthetase (FACS) successfully generated four novel leads exhibiting high binding affinity and dynamic stability (Pawar et al., 2025). For pantothenate synthetase (PS) and Mtb topoisomerase I (Mttopo I), optimized descriptor combinations coupled with Bayesian modeling and molecular docking yielded high-precision predictive models, facilitating the identification of corresponding lead compounds and establishing proof-of-concept for these targets in anti-TB drug development (Ekins et al., 2017; Hass et al., 2022). ML further bridges hit identification and polypharmacology; integrated ML–MD workflows identified dual-targeting candidates-including DB11753 and DB14703-against DNA gyrase A (GyrA) and serine/threonine protein kinase B (PknB), validating the efficiency of data-driven approaches for cross-target repurposing (Lee et al., 2024)**.** Addressing the critical challenge of poor mycobacterial cell wall permeability, researchers constructed a high-performance artificial neural network model (AUC = 0.911) using fragment descriptors derived from >5,000 compounds, providing a computational tool to overcome limited intracellular drug accumulation (Radchenko et al., 2023). In the domain of resistance prediction, gradient boosting classifiers (GBC) achieve up to 97.28% accuracy in predicting drug resistance from whole-genome sequencing (WGS) data; integration of SHAP values quantifies the contribution of key resistance-determining loci (e.g., *rpoB* and *katG*) markedly enhancing model interpretability (Xu et al., 2025; Yang et al., 2018). A global analysis of 13,402 clinical isolates from 16 countries further demonstrated that logistic regression and gradient tree boosting, when integrated with dimensionality reduction techniques, outperform conventional diagnostics (AUC improvements >23% for specific drugs) and facilitate the discovery of novel resistance markers (Kouchaki et al., 2019). Complementing these advances, a rapid, culture-free detection strategy combining Raman spectroscopy with ML achieves >98% accuracy in drug-resistance classification from dried samples, with clinical deployability validated using low-cost portable devices in resource-limited settings (Ogunlade et al., 2024).

In therapeutic drug monitoring (TDM) and clinical decision support, ML is driving the translation of precision medicine into routine TB care. For isoniazid, ML models incorporating demographic, genotypic, and clinical covariates enable high-precision prediction of 2-h plasma concentrations (C_2_h), achieving 93.4% accuracy and improving target attainment rates by 43.7% compared with conventional approaches (Tang et al., 2024). In the context of MDR/rifampicin-resistant TB (MDR/RR-TB), artificial neural networks predict sputum culture conversion at 2 and 6 months with AUCs of 0.82 and 0.90, respectively, offering an early indicator of therapeutic efficacy (Zhang et al., 2025). Concurrently, models trained on routine surveillance data concurrently predict fluoroquinolone resistance at diagnosis, thereby informing treatment initiation while awaiting phenotypic susceptibility results (You et al., 2022).

Beyond surrogate endpoints, comparative analyses of multiple ML algorithms identified decision trees as the optimal framework for predicting pulmonary TB treatment response, achieving 92.72% accuracy and an AUC of 0.909 (Ahamed Fayaz et al., 2024). Expanding into decision support, ML-driven clinical decision support systems (CDSS)-developed by quantifying drug and regimen characteristics and refined through expert feedback-demonstrate considerable potential to optimize outcomes in patients with difficult-to-treat drug-resistant TB, even amid operational constraints such as drug stockouts and guideline non-adherence (Verboven et al., 2024). Collectively, these advances illustrate that ML has reshaped the anti-TB drug development continuum-from hit identification to clinical optimization. Representative applications include OCHEM-platform–based predictive modeling of isoniazid derivatives (Kovalishyn et al., 2020) and Bayesian literature mining to prioritize compounds for *in vitro* validation (Lane et al., 2018).Together, this evidence signals a paradigm shift: TB research is transitioning from empirical trial-and-error toward precise, intelligent, data-driven frameworks that offer robust technical support for conquering drug-resistant TB (Table 3).

**TABLE 3:** Summary of ML applications in Anti-TB drug discovery and development.

Main application directions	ML method	Targets/Signaling pathways	Therapeutic applications	References (Ref.)
Screening of Lead Compounds and Repurposed Old Drugs	SVM, Deep Learning	DNA gyrase	Aldoxorubicin and Quarfloxin	(Yao et al., 2023)
	SVM	Thymidylate kinase	Top three candidate compounds:ZINC1360209602, ZINC1435770877, ZINC1456128327	(Rawat et al., 2025)
	RF, XGBoost, DT, KNN, GNB, LR, ANN	CHEMBL360, CHEMBL2111188, CHEMBL612960, CHEMBL613086 and CHEMBL615052	Moxifloxacin (MOX) (Morgan 497 and Morgan 650, Delamanid (Morgan 530 and Morgan 317)	(Fawibe et al., 2025)
	DNN/*De novo* design	Fatty Acyl-CoA synthetase protein of Mtb	(E)-6-methoxy-3-styryl-7-((3,4,5-trihydroxy-6-(hydroxymethyl) tetrahydro-2h-pyran-2-yl) oxy)-2h-chromen-2-one	(George et al., 2026)
	Bayesian modeling	Mttopo I	For PS and Mttopo I, by integrating descriptor optimization, Bayesian modeling and molecular docking, high-precision models were constructed for lead compound screening, and their druggability was validated	(Hass et al., 2022)
	ANN, GBR, RFR, GPR	Pantothenate synthetase	2-(2-formamidobenzamido)-3-(1H-indol-3-yl) propanoic acid N-formyl-3-(5-methylene-4-oxo-2-thioxothiazolidin-3-yl) propanehydrazide; imidazo [2,1-b] thiazole and benzo [d]imidazo [2,1-b] thiazole 5-tert-Butyl-N-pyrazol-4-yl-4,5,6,7-tetrahydrobenzo [d]isoxazole-3-carboxamide 3-phenyl-4, 5, 6, 7-tetrahydro-1H-pyrazolo [4,3-c] pyridine	(Radchenko et al., 2023)
	RF + Docking	GyrA and PknB	DB11753 and DB14703	(Dos Santos et al., 2021)
	LR, SVM, RF, PM, CBMM	rpoB and katG et al.	isoniazid (INH), rifampicin (RIF), Ethambutol (EMB), Pyrazinamide (PZA), Ciprofloxacin (CIP), MOX, ofloxacin, streptomycin	(Kouchaki et al., 2019)
	GBC + SHAP	rpoB and katG	INH, RIF, PZA, and EMB	(Wu et al., 2020)
	LR, GBT + Dimensionality Reduction	rpoB and katG et al.	INH, RIF, OFX, CAP, EMB, PZA, Amikacin (AK), CIP, Kanamycin (KAN) and MOX	(Niazi, 2025)
	Lightweight ML + Raman	katG	INH, RIF, MOX and AK	(Tang et al., 2024)
Clinical Therapeutic Drug Monitoring and Efficacy Evaluation	ANN	Mycobacterial Cell Wall Permeability	Based on ANN and fragment descriptors, a prediction model (AUC = 0.911) was constructed using a permeability dataset of more than 5000 compounds, providing a key computational tool for improving the intracellular activity of drugs	(Ogunlade et al., 2024)
	CatBoost	NAT2 genotypes, weight and race	A ML model was developed with robust predictive performance, enabling the determination of individualized initial doses of isoniazid​ for adult patients	(Zhang et al., 2025)
	ANN	Demographic information, clinical data, and sputum culture conversion outcomes at 2 months post-treatment initiation	ANN achieves the optimal prediction of 2- and 6-month culture conversion outcomes in MDR/RR-TB patients (AUC = 0.82/0.90), facilitating early therapeutic efficacy evaluation	(You et al., 2022)
	LR, RF,DNN	demographic and TB clinical information of patients and the estimated district-level prevalence of resistance	Models were constructed using routine surveillance data to predict fluoroquinolone resistance at the time of diagnosis, assisting medication decision-making during the drug susceptibility testing waiting period	(Ahamed Fayaz et al., 2024)
	Decision Tree	sputum culture conversion	The decision tree was identified as the optimal model for predicting sputum culture conversion in pulmonary TB, achieving an accuracy of 92.72% and an AUC of 0.909, which supports clinical therapeutic efficacy evaluation	(Verboven et al., 2024)

### 2.3 Primary screening of microscopic binding modes and dynamic refined validation: from molecular docking to MD simulation

Although network pharmacology identifies core therapeutic targets, it cannot resolve atomic-level binding determinants, thereby necessitating high-precision structural biology approaches. Molecular docking-a core virtual screening technique predicated on receptor three-dimensional structure-predicts the optimal binding pose and affinity between small-molecule ligands and macromolecular receptors (Meng et al., 2011). In anti-infective drug discovery, docking has proven instrumental in deciphering mechanisms of action and prioritizing lead candidates. For human African trypanosomiasis, integrated docking and quantitative structure–activity relationship (QSAR) analyses revealed that active Pathogen Box compounds inhibit phosphoglycerate kinase/glyceraldehyde-3-phosphate dehydrogenase (PGK/GAPDH) through electrostatic surface complementarity and favorable atomic packing, thereby facilitating the discovery of novel antitrypanosomal agents (Ogunleye et al., 2021). In parallel, docking studies on Chagas disease elucidated the covalent binding mechanism of benzenesulfonylhydrazone derivatives to cruzain, clarifying the molecular basis for their anti-*Trypanosoma cruzi* activity (Elizondo-Jimenez et al., 2017). More recently, in anti-HIV drug design, docking has mapped the key residue interactions between colchicine derivatives and the viral entry protein gp120, guiding the rational design of novel inhibitors when coupled with QSAR modeling (Worachartcheewan et al., 2019). Furthermore, docking integrated with three-dimensional QSAR (3D-QSAR) and MD simulations has directed the development of highly potent biphenyl-substituted pyridinone non-nucleoside reverse transcriptase inhibitors targeting HIV-1 reverse transcriptase (Jiang et al., 2024).

Confronting the escalating threat of drug-resistant tuberculosis, molecular docking has become indispensable across the entire anti-TB drug discovery pipeline—from target validation to hit optimization and drug repurposing. In the realm of drug repurposing, docking integrated with ML-based virtual screening confirmed the direct binding of doxorubicin and quinolones to Mtb DNA gyrase, providing robust evidence for their clinical repositioning (Zheng et al., 2024). Similarly, integrated workflows combining docking, ADMET profiling, and MD simulations identified 5,7-dihydroxydehydroiso-α-lapachone as a potent inhibitor of InhA—a key enzyme in mycolic acid biosynthesis—exhibiting a superior binding affinity (−9.5 kcal/mol) and enhanced dynamic stability compared with isoniazid (Fawibe et al., 2025). For hit-to-lead optimization, molecular docking has extensively characterized the binding modes of novel chemotypes targeting InhA (enoyl-ACP reductase). Representative series include 4-(1,2,3-triazolyl) aryl ketones (Turkmen et al., 2022), indolizidine derivatives (Venu et al., 2021), Quinoxaline–oxadiazole hybrids (Kanchrana et al., 2024), benzimidazole–piperidine imines (Revathi et al., 2015) and N′-arylmethylene-4-nitrobenzoylhydrazide (Bhosale et al., 2022) etc. Collectively, these studies delineated SARs that correlated strongly with *in vitro* bioactivities. Further mechanistic clarity was provided by docking analyses of fused pyrimidine-5-carbonitrile derivatives (Xia et al., 2025) and 1,2,3-/1,2,4-triazole hybrids (El Sawy et al., 2022), which revealed potent target occupancy within the InhA active site.

Regarding drug resistance mechanisms, docking elucidated the binding of modified ethambutol analogs to arabinosyltransferase C (EmbC), revealing the molecular basis by which *Emb1* and *Emb3* may reverse resistance (Das et al., 2020). Concurrently, docking of quinazolinone analogs against the LfrA efflux pump clarified their potential to inhibit drug export, thereby restoring intracellular drug accumulation (Sen et al., 2018). In the context of multi-target strategies, docking coupled with experimental binding assays identified riboflavin analog 5a​ as a selective growth inhibitor (MIC = 6.25 µM) that targets the FMN riboswitch to suppress riboflavin biosynthesis, offering a high-selectivity, low-cytotoxicity therapeutic avenue (Harale et al., 2021). Furthermore, by integrating docking, MD simulations, and essential dynamics, engineered NKLF2 mutants (M16C/M16I) exhibited enhanced polypharmacology against essential Mtb enzymes—including EmbC, DNA gyrase, Rv1414c (S1), and InhA—effectively circumventing resistance through simultaneous target inhibition (George et al., 2026). Beyond efficacy, docking contributes to safety and optimization. Network toxicology integrated with docking identified NFE2L2, MAPK14, and NFKB1 as hub targets mediating anti-TB drug-induced hepatotoxicity via oxidative stress and apoptosis, suggesting strategies to mitigate toxicity (Liu et al., 2025). Addressing the excessive lipophilicity of bedaquiline, docking-guided rational design yielded novel derivatives with improved physicochemical properties while maintaining high-affinity binding to ATP synthase (Wu et al., 2020). In summary, molecular docking constitutes a comprehensive workflow encompassing virtual screening, hit identification, binding mode analysis, and SAR establishment. Its irreplaceable value in overcoming resistance, enabling multi-target drug design, and facilitating drug repurposing is now firmly established (Table 4).

**TABLE 4:** Applications of molecular docking in Anti-TB drug development.

Main application directions	Typical research contents/cases	Targets/Signaling pathways	Candidate drugs/Clinical application	References (Ref.)
Virtual Screening and Drug Repurposing	Doxorubicin and quinolone directly target the DNA gyrase of Mtb	DNA gyrase	aldoxorubicin and quarfloxin	(Yao et al., 2023)
	For InhA, molecular docking was used to verify the binding modes of various novel derivatives	enoyl-acyl carrier protein (ACP)-reductase (InhA)	5,7-dihydroxydehydroiso-α-lapachone	(Kanchrana et al., 2024)
		enoyl-acyl carrier protein reductase enzyme	4-(1,2,3-triazoyl) arylmethanone derivatives	(Venu et al., 2021)
		InhA	indolizine derivatives	(Revathi et al., 2015)
	Spiroquinoxaline-1,2,4-oxadiazole derivatives​ have demonstrated significant anti-mycobacterial potential	The Mtb protein 5OEQ	spiroquinoxaline-1,2,4-oxadiazole derivatives	(Xia et al., 2025)
	These findings provide valuable insights for the further development of optimized anti-TB agents, highlighting piperidin-4-imine derivatives​ as promising candidates targeting EACP reductase	Enoyl-ACP reductase (FabI/InhA)	piperidin-4-imine derivatives	(Bhosale et al., 2022)
	Current study proposes N′-arylidene-4-nitrobenzohydrazides as anti-TB agents. Agents with such system can be developed in future for development into active lead molecules	1ENY,1TED and 2FUM	N'-arylidene-4-nitrobenzohydrazides	(El Sawy et al., 2022)
	For fused pyrimidine-5-carbonitrile and triazole hybrids, molecular docking elucidated the potent target-occupying mechanism of their ultra-highly active inhibitors	InhA (NADH-dependent enoyl-ACP reductase)	pyridin-2-yl-pyrimidine	(Valencia et al., 2022)
		InhA (NADH-dependent enoyl-ACP reductase)	Hybrid 1,2,4- and 1,2,3-Triazoles	(Das et al., 2020)
Analysis of drug resistance mechanisms	To analyze the interaction between ethambutol-modified molecules and EmbC, and reveal the molecular basis of Emb1/Emb3 as drug resistance reversers	Arabinosyltransferase C	Ethambutol, isoniazid, and their structurally modified derivatives	(Sen et al., 2018)
	Calculate the interaction between quinazolinone analogues and LfrA, and clarify their potential to reverse drug resistance as efflux pump inhibitors	LfrA	11H-pyrido [2,1-b] quinazolin-11-one analogues	(Harale et al., 2021)
Multi-target exploration	To calculate the binding of riboflavin analogs to the FMN riboswitch, reveal the anti-TB mechanism, and identify the lead compound 5a	Flavin mononucleotide (FMN) riboswitch	Riboflavin derivatives	(Liu et al., 2025)
	The antimicrobial peptide mutant (NKLF2_M16C/I) was docked with multiple targets such as EmbC and DNA gyrase	EmbC, DNA gyrase, 30S ribosomal protein S1, and enoyl-[acyl-carrier-protein]	NKLF2 and its mutants (M16C and M16I)	(Hanwarinroj et al., 2022)
Safety evaluation and structural optimization	Molecular docking was performed to evaluate the safety of anti-TB drugs, and to reveal the potential mechanism underlying hepatotoxicity induced by their binding to NFE2L2/MAPK14	NFE2L2, NFKB1, MAP2K1, MAPK14, IGF1R and GSK3B	isoniazid, rifampicin, pyrazinamide	(Guterres and Im, 2020)
	In view of the excessive lipophilicity of bedaquiline, molecular docking guided the design of low-lipophilic derivatives and elucidated their binding mechanism to ATP synthase	ATP synthase	Bedaquiline	(Shahbaaz et al., 2022)

MD simulation is a computational technique rooted in classical Newtonian mechanics that numerically solves atomic equations of motion to monitor the time-dependent behavior of molecular systems at atomic resolution. As such, MD serves as a rigorous mechanistic validation tool, enabling real-time tracking of complex evolution and quantitative binding affinity estimation through free-energy calculations. Methodologically, MD approaches form a continuum balancing computational cost against reliability: First, classical MD (cMD)​ employs all-atom force fields to assess complex stability (e.g., via RMSD and RMSF analyses) (Guterres and Im, 2020); while computationally affordable, it often fails to capture rare events or provide precise thermodynamic/kinetic quantification. Second, enhanced sampling techniques—such as umbrella sampling and metadynamics-explore binding pathways and kinetic barriers (Stratmann et al., 2025), overcoming sampling bottlenecks at higher computational expense and requiring expert-defined collective variables, though absolute accuracy remains parameter-dependent. Third, free-energy methods range from endpoint estimates (MM/PBSA) for hit ranking to alchemical calculations (free energy perturbation [FEP], thermodynamic integration [TI]) for high-precision affinity prediction during lead optimization (Yang M. et al., 2023; Hahn et al., 2024). Although regarded as the “gold standard,” these methods incur the highest computational cost, are constrained by force-field limitations, and are typically confined to late-stage optimization rather than large-scale screening. These tiers are frequently integrated into hierarchical workflows (e.g., Docking → cMD → FEP) to balance efficiency and accuracy (Jacobsen et al., 2023). Beyond methodology, MD has been deeply embedded in broad-spectrum anti-infective drug discovery. In antiviral research, MD simulations have validated the stable binding and dynamic recognition of small molecules targeting critical SARS-CoV-2 proteins, including RNA-dependent RNA polymerase (RdRp) and the main protease (Mpro) (Alturki et al., 2022; Tanimoto et al., 2022; Komatsu et al., 2020), and have elucidated conformational rearrangements induced by resistance mutations in essential HIV enzymes such as protease and integrase (Rana et al., 2022; Chen et al., 2015). Regarding bacterial and parasitic infections, MD has illuminated the membrane-disrupting mechanisms of antimicrobial peptides (Palmer et al., 2021) and the inhibitory dynamics of compounds targeting *Streptococcus pneumoniae* RNA polymerase (Ye et al., 2022). Crucially, MD plays a central role in combating drug resistance: it unravels the molecular consequences of resistance mutations in the Gram-negative metallo-β-lactamase NDM-1 (Rehman et al., 2019) and PBP2x (Ge et al., 2012), while guiding the rational design of novel inhibitors capable of overcoming resistance mediated by the influenza A/M2 proton channel (Wang et al., 2011) and *Klebsiella pneumoniae* LpxC (Ahmad et al., 2019).

Within the field of anti-TB drug discovery, molecular dynamics (MD) simulation has emerged as an indispensable core technique owing to its capacity to resolve the dynamic behavior of biological macromolecules and drug–target interactions at atomic resolution. Current applications predominantly center on four domains: elucidating target-binding mechanisms, dissecting drug resistance, investigating biomembrane interactions, and implementing integrated virtual screening workflows.

First, regarding target-binding elucidation and lead optimization, MD simulations extensively characterize the dynamic binding modes of candidate compounds against essential Mtb targets. Integrated workflows combining MM-PBSA/MM-GBSA free-energy calculations with Density Functional Theory (DFT) optimization have validated the thermodynamic stability of diverse novel chemotypes. Representative examples include ZINC000252693842 bound to RNA polymerase (Fakih et al., 2025), benzimidazole derivatives (1p, 1q, 1t) with the PrpR protein (Rajasekhar et al., 2021), and quinolinone-thiosemicarbazone derivatives (11days, 11e) interacting with key enzymes, several of which exhibit superior *in vitro* activity compared to isoniazid (Valencia et al., 2022). Notably, a high-throughput virtual screening workflow incorporating DFT and MD identified polyketide synthase 13 (Pks13) and PknG inhibitors from microbial secondary metabolites, demonstrating that synthetic optimization of vazabitide A significantly enhances its binding affinity for Pks13, thereby providing novel scaffolds for drug development (Nyambo et al., 2024). Mechanistic insights further reveal that 4-amino-1,2,4-triazole derivatives lock the catalytic conformation of β-ketoacyl-ACP synthase I (KasA) over 200-ns trajectories (Venugopala et al., 2020) while Tam16 inhibits Pks13 via a distinctive hydrogen-bonding network (Cruz et al., 2019). Additionally, nickel-based Schiff bases enhance the structural rigidity of class A β-lactamase (BlaC) (Junaid et al., 2018), and quinazoline derivatives achieve high-affinity binding to protein kinase B (PknB) through critical interactions with Glu93 and Val95, guiding the rational design of the optimized lead D060 (Hanwarinroj et al., 2022). Other studies have characterized the stable binding of thiazolidine derivatives (1k, 1j) to PrpR (Rajasekhar et al., 2022), identified key pharmacophoric residues (Glu93, Val95, Leu17) in PknB targeted by aminopyrimidine inhibitors (Punkvang et al., 2015), and confirmed the favorable binding properties of thiadiazole derivatives AK05 and AK13 against the hydroxysteroid dehydrogenase-like protein A (HsaA) monooxygenase (Zheng et al., 2025).

Second, in targeting membrane and cytoskeletal proteins, MD simulations have uncovered unique inhibitory mechanisms. For the outer membrane protein OmpATb, simulations demonstrate that natural products-exemplified by ZINC000003958185—occupy the hydrophobic channel lumen, providing a structural rationale for designing inhibitors that potentiate the cellular uptake of hydrophilic antibiotics (Kwofie et al., 2020). Targeting the cytoskeleton, integrated MD and density functional theory (DFT) calculations revealed that fusidic acid (FusA) forms a stable complex with filamenting temperature-sensitive mutant Z (FtsZ), restricting its conformational flexibility and inhibiting polymerization more effectively than citric acid (Akinpelu et al., 2020). Against the DNA gyrase B subunit, virtual screening and structural optimization identified novel pyrroloquinazoline derivatives (PQPNN) as promising candidates that stably bind the ATPase domain (Arevalo and Amorim, 2022).

Third, MD simulations are pivotal in dissecting drug resistance mechanisms.​ By comparing wild-type and mutant dynamics, studies demonstrated that *katG* Met255Ile and Ala222Arg mutations destabilize the enzyme and attenuate isoniazid binding, thereby conferring resistance (Shahbaaz et al., 2022). Enhanced sampling via Gaussian accelerated MD (GaMD) further revealed that *rpoB* S456L/D441V mutations disrupt the hydrogen-bond network between R454 and rifampicin, triggering outward drug displacement and weakening target engagement (Zhang et al., 2020).

Fourth, in membrane systems and synergistic drug effects, MD coupled with spectroscopy elucidated how bioactive compounds modulate the Mtb inner membrane. Both benzohydroxamic acid (BH) and nitrobenzohydroxamic acid (NBH) induced global membrane ordering by increasing bilayer thickness (Kokuszi et al., 2024), whereas mefloquine (MFL) enhanced lateral diffusion and permeability, clarifying the biophysical basis for its synergy with first-line agents (Dos Santos et al., 2021). Finally, multi-method integration has substantially improved virtual screening accuracy. Combining MD with molecular docking, ML, and transcriptomics has enabled the identification of multiple leads, including NPC41982 (targeting AccD6) (Singha and Krishna, 2025), specific hit inhibitors against GyrB (Kashyap et al., 2019), and β-lactam lead compounds acting on the L, D-transpeptidase five enzyme (Sabe et al., 2019). Meanwhile, quantitative models established based on conformational analysis of the InhA enzyme–ligand complex can account for 80% of the biological activity variance (da Silva Lima et al., 2015). Complementing these efforts, studies on malic enzyme (MEZ) conformational flexibility under substrate binding (Burley et al., 2021), alongside research characterizing the stable binding of medicinal plant-derived compounds (e.g., physalin D) to L-asparaginase (Sharma et al., 2023), further enrich and expand the strategic framework for anti-TB drug development. Collectively, MD simulations have evolved from a conformational sampling tool into an indispensable core technology spanning the full anti-TB drug discovery workflow-encompassing mechanistic interpretation, virtual screening, and resistance elucidation (Table 5).

**TABLE 5:** Summary of the applications of MD simulations in anti-TB drug development.

Main application directions	Typical research contents/cases	Targets/Signaling pathways	Candidate drugs/Clinical application	References (Ref.)
Analysis of Target Binding Mechanism and Lead Compound Optimization (enzyme and protein targets)	MM-PBSA/GBSA combined with DFT were used to verify the binding stability of ligands to various enzymes and proteins. Compounds including ZINC252693842, benzimidazoles, and quinolinone thiosemicarbazones exhibited strong binding affinity, and some of them showed better activity than isoniazid	RNA polymerase	ZINC001286671821, ZINC000253654686 and ZINC000252693842	(Rajasekhar et al., 2021)
	Molecular docking and dynamics simulations identified benzimidazole derivatives (1p, 1q, 1t) that bind precisely to MtbPrpR (ARG308, etc.), exerting superior anti-TB activity compared to isoniazid via targeted stabilization of the regulator	ARG308, GLY189, VAL312, LEU403, and LEU190 amino acid residues of MTB PrpR	Benzimidazole derivatives	(Nyambo et al., 2024)
	Computational design and MD simulations confirm that quinolinone-thiosemicarbazones​ potently inhibit InhA and DprE1, thereby disrupting cell wall biosynthesis and energy metabolism to exert superior anti-TB activity over isoniazid and ofloxacin	enoyl-acyl carrier protein reductase (InhA) and decaprenylphosphoryl-β-D-ribose-2′-oxidase (DprE1)	Novel quinolinone-based thiosemicarbazones	(Venugopala et al., 2020)
	DFT optimization revealed that cyclic peptide derivatives such as vazabitide A form highly stable complexes with Pks13/PknG	Pks13/PknG	cyclo-(L-Pro-4-OH-L-Leu) and vazabitide A	(Cruz et al., 2019)
	200 ns MD simulations confirmed that 4-amino-1,2,4-triazole-3-thiol derivatives specifically lock the catalytic conformation of KasA, exhibiting better activity than thiolactomycin	KasA	4-amino-5-(4-fluoro-3-phenoxyphenyl)-4H-1,2,4-triazole-3-thiol	(Pawar et al., 2025)
	Elucidate the mechanism by which Tam16 inhibits Pks13 through hydrogen bond networks	Pks13	Tam1 and its analogs	(Junaid et al., 2018)
	Nickel-based Schiff bases suppress BlaC via structural rigidification	β-lactamase (BlaC)	Nickel-Schiff base complexes	(Rajasekhar et al., 2022)
	Quinazoline derivatives stabilize PknB through Glu93/Val95 interactions to inhibit the enzyme, thereby guiding the rational design of compound D060	PknB	Novel quinazoline-based compounds	(Kashyap et al., 2019)
​	It covers the stable binding of thiazolidine derivatives (1k/1j) to PrpR, the recognition interaction between aminopyrimidine compounds and PknB via Glu93/Val95/Leu17 residues, as well as the binding superiority of thiadiazole derivatives AK05 and AK13 on HsaA	PrpR	Thiazolidine derivatives (1k/1j)	(Punkvang et al., 2015)
	MD simulations and binding free-energy calculations elucidate that substituted aminopyrimidine inhibitors exert anti-TB effects by specifically binding to MtbPknB (via Glu93, Val95, Leu17) and blocking kinase activity through key interactions of the aminopyrazole and pyrimidine cores	PknB	Aminopyrimidine compounds	(Zheng et al., 2025)
	Molecular docking and dynamics simulations confirm that AK05 and AK13​ stably bind to MtbHsaA monooxygenase, disrupting cholesterol metabolism to exert anti-TB effects with superior binding affinity and drug-likeness compared to GSK2556286.”	HsaA	Thiadiazole derivatives	(Kwofie et al., 2020)
Analysis of Target Binding Mechanism and Lead Compound Optimization (Targeted Research on Membrane Proteins and Structural Proteins)	Confirmed that natural products such as ZINC3958185 occupy the hydrophobic channel of OmpATb	OmpATb	Natural products such as ZINC3958185	(Arevalo and Amorim, 2022)
	Combined with DFT verification, fusidic acid (FusA) forms a stable complex with FtsZ and restricts its conformational flexibility	FtsZ	Fusidic acid	(Sharma et al., 2023)
	Novel PQPNN compounds were obtained via virtual screening, which were verified to stably target the subunit B of DNA gyrase	DNA gyrase	Pyrrolo [1,2-a] quinazoline derivative	(Kokuszi et al., 2024)
Elucidation of Drug Resistance Mechanism	Dynamic comparison between wild-type and mutant strains revealed that the KatG M255I/A222R mutations induce drug resistance by impairing protein stability and reducing the binding affinity of isoniazid	KatG M255I/A222R	Isoniazid	(Zhang et al., 2020)
	GaMD further revealed that the S456L/D441V mutations of RNA polymerase disrupt the hydrogen bond network between R454 and rifampicin	S456L/D441V mutations of RNA polymerase	Rifampicin	(Luan et al., 2025)

## 3 Construct a multi-scale closed-loop framework combining the “top-down” and “bottom-up” approaches

Systems pharmacology, network pharmacology, ML, molecular docking, and MD simulations each exhibit unique advantages in the research and development of anti-TB drugs, as for Figure 2. However, a single method is often limited by the “flux-precision” trade-off. Therefore, constructing a cross-scale computational workflow has become the current mainstream trend, aiming to achieve full-chain coverage from macro network mechanisms to micro atomic interactions. A typical integration strategy follows a combined “top-down” and “bottom-up” approach. Validation not only overcomes the limitations of a single method but also provides a systematic solution for addressing the drug resistance of Mtb. The multi-dimensional validation framework can effectively address the inherent limitations of individual computational simulation methods and provide a systematic solution for drug discovery against drug-resistant *Mycobacterium tuberculosis*. To operationalize this core proposition, the experimental and computational validation of multi-scale predictions can be systematically organized into four complementary technical tiers: *in vitro* validation, *in vivo*/preclinical validation, clinical/translational validation, and computational cross-validation coupled with multi-omics profiling. Each tier specifically targets the technical blind spots of individual methods. Ultimately, through an integrated workflow rather than the isolated application of any single method, anti-tuberculosis lead compounds with both mechanistic rationality and experimental reliability can be identified. *In vitro* activity assays serve as the primary validation gate following the preliminary screening of candidate compounds via molecular docking and molecular dynamics simulations. Compounds with favorable docking scores but no minimum inhibitory concentration (MIC) activity can be directly eliminated at this stage, thereby effectively reducing the false-positive rate of virtual screening and avoiding futile investment of subsequent R&D resources (Xia et al., 2025; Venu et al., 2021; Bhosale et al., 2022). *In vivo* pharmacodynamics and pharmacokinetics (PK) studies further fill the gap in drug distribution and physiological efficacy information that cannot be captured by atomistic simulations. They delineate the inhibitory effect of candidate compounds on *M. tuberculosis* burden in living systems, providing physiological-level experimental support for druggability assessment (George et al., 2026). Clinical validation constitutes the core terminal link in the closed-loop R&D pipeline, with its core value lying in confirming that computationally identified target vulnerabilities can be effectively translated into clinical patient benefits. Exemplified by the ATP synthase (*atpE*) target and the approved agent bedaquiline, computationally predicted drug targets are ultimately validated in clinical practice (Kumar et al., 2015; Janssen et al., 2025). Beyond stepwise experimental validation, computational cross-validation mitigates bias introduced by individual algorithms at the methodological level, effectively avoids the risk of model overfitting, and ensures the robustness and reproducibility of prediction results (Zheng et al., 2024). Multi-omics profiling, via independent experimental measurement data, provides empirical evidence for the authentic target engagement of predicted targets in pathogens or hosts, further consolidating the experimental foundation of target mechanisms of action (Suresh et al., 2025; Singh and Purohit, 2025). Collectively, the aforementioned tiers form a progressive evidence chain, with each step offsetting the inherent limitations of the preceding computational simulation layer. Through the systematic integration of multi-dimensional validation, the constructed drug discovery strategy for drug-resistant tuberculosis features both a solid mechanistic foundation and robust experimental evidence support, offering a reliable and systematic solution for novel drug development against drug-resistant *M. tuberculosis*.

**FIGURE 2:**
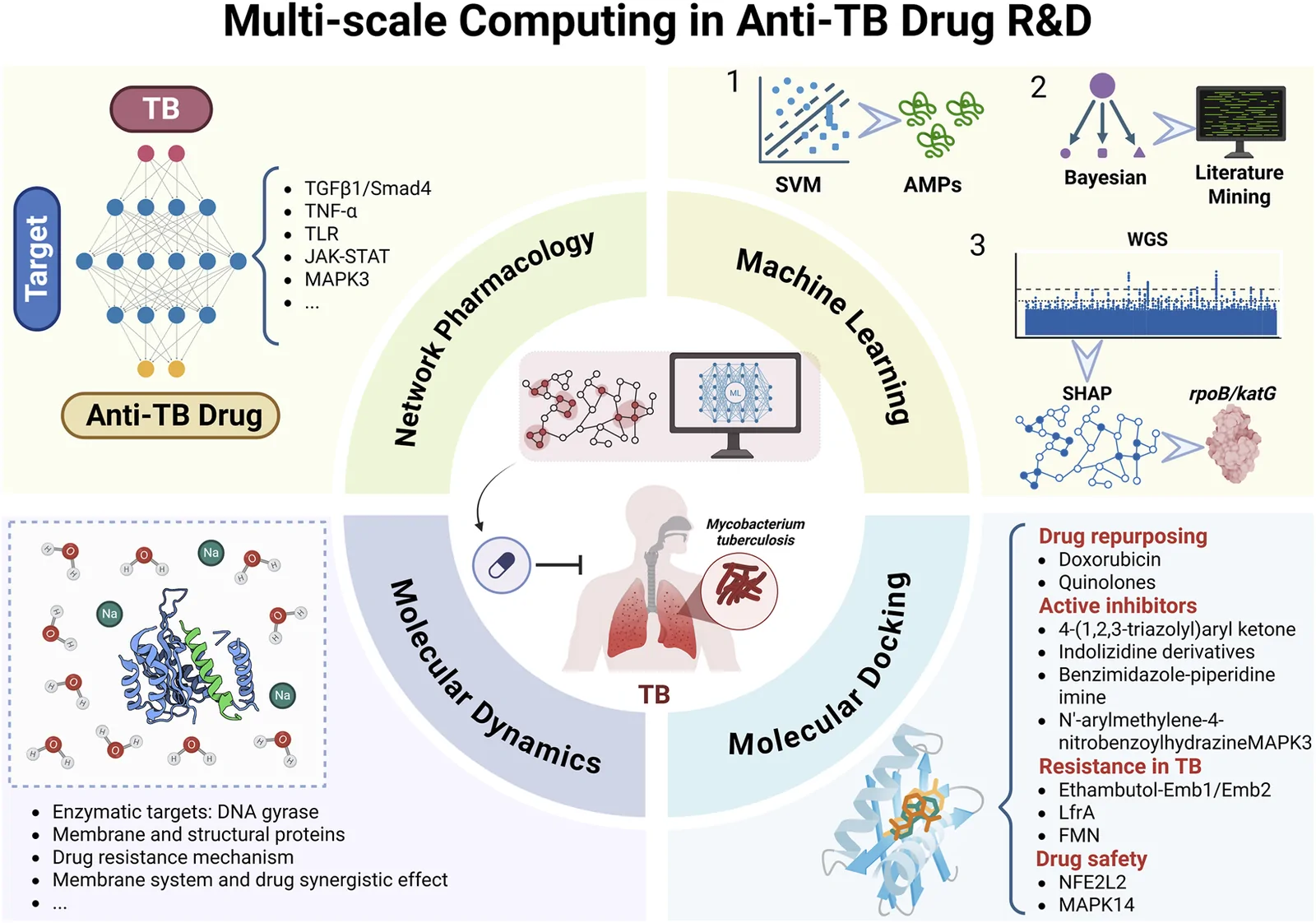
Multi-scale computing in Anti-TB durg R&D.

Therefore, these computational layers are not merely additive but synergistic: systems/network pharmacology defines the biological “stage,” ML accelerates the search for molecular “actors,” and MD confirms the precise “performance” at the atomic level. This hierarchical integration constitutes a closed-loop framework essential for overcoming the inherent limitations of any single method in anti-TB drug discovery. Technically, this vertical integration is achieved through bidirectional data flow. Downward mapping​ translates network-derived nodes (e.g., differentially expressed genes or enriched pathways) into structural targets for molecular docking and dynamics simulations, ensuring that atomic-level studies are biologically grounded rather than randomly selected. Conversely, upward validation​ feeds the energetic and conformational outputs of MD simulations (e.g., binding free energy, allosteric changes) back into the network model to refine the predicted phenotypes or identify off-target effects. This bidirectional linkage ensures conceptual coherence across scales.

Despite substantial advances in multi-scale computational methodologies, several persistent challenges limit their practical translation into anti-tuberculosis (anti-TB) drug discovery pipelines. First is data scarcity and suboptimal data quality. Relative to oncology or HIV research, the repertoire of high-resolution crystal structures of Mtb remains comparatively limited, and the three-dimensional structures of many key targets—such as certain membrane proteins—have yet to be resolved. This deficit impedes the advancement of structure-based drug design (SBDD) and compromises the training accuracy of machine learning (ML) models. Second is the dynamic complexity of drug-resistant mutations. The molecular mechanisms underlying Mtb drug resistance are highly intricate, frequently involving combinatorial gene mutations and efflux pump overexpression. Currently, most MD simulations are confined to characterizing the effects of single amino acid substitutions (e.g., Met255Ile in KatG) (Shahbaaz et al., 2022), making it difficult to comprehensively recapitulate the authentic physiological context of multidrug-resistant (MDR) strains at the atomic level. Third is the inherent trade-off between computational accuracy and resource cost. While approaches such as quantum mechanics/molecular mechanics (QM/MM) deliver exceptional precision at the electronic level, their prohibitive computational overhead restricts deployment in large-scale virtual screening campaigns. Conversely, rapid docking algorithms commonly disregard protein backbone flexibility, which may introduce false-positive hits in candidate prioritization. Finally, a notable translational gap persists between *in vitro* predictions and *in vivo* efficacy. Most computational simulations are parameterized under *in vitro* biochemical conditions and fail to fully recapitulate the complex *in vivo* host–pathogen microenvironment—for instance, the hypoxic and acidic niches within granulomas—leading to discrepancies between computational outputs and actual drug performance. Beyond these technical barriers inherent to individual methods, the integration of multi-scale methodologies itself introduces distinct challenges that merit careful consideration. First, dimensional incompatibility is a pervasive issue: network pharmacology operates on abstract nodes and edges at the systems level, whereas MD simulations demand explicit atomic coordinates. Translating high-level network perturbations into physically meaningful structural modifications suitable for MD simulation remains largely heuristic, rather than governed by standardized algorithmic workflows. Second, cross-scale error propagation poses a substantial risk. Biases or false-positive hits generated during high-throughput ML screening are inevitably propagated into downstream MD validation steps, accelerating a “garbage in, garbage out” dynamic that erodes the reliability of the entire pipeline. Third, interpretability and reproducibility are diminished when black-box ML models are combined with complex network topologies. This opacity not only makes it exceedingly difficult for medicinal chemists to derive actionable chemical insights, but also impedes replication of computational findings by independent laboratories in the absence of standardized protocols. Addressing these integration-specific challenges is therefore critical to advancing multi-scale frameworks from theoretical constructs into functional drug discovery pipelines. In addition, while the sequential “Network → ML → Docking → MD” framework is theoretically compelling, systematic end-to-end benchmarking studies validating this complete pipeline specifically in the TB field remain scarce. Current literature predominantly features modular applications rather than unified, integrated workflows. For example, network pharmacology has been successfully applied to map *M. tuberculosis* protein–protein interaction (PPI) networks and identify therapeutic targets through topological analysis (e.g., via STRING and Cytoscape) (Luo et al., 2024), yet such studies rarely extend downstream to compound screening. Conversely, ML integration has demonstrated efficacy in optimizing docking accuracy (e.g., MILCDock) and accelerating virtual screening via pre-filtering strategies (Morris et al., 2022; Luttens et al., 2025), but these approaches typically operate independently of upstream network-level predictions. MD continues to serve as the gold standard for validating docking poses and evaluating binding stability (Ballabh et al., 2025; Stelitano et al., 2025), functioning as a downstream filtering step rather than an integral component of a closed-loop workflow. Tools such as MILCDock and iMiner, alongside large-scale resources including the MolData dataset, demonstrate the technical feasibility of deploying these individual models (Morris et al., 2022; Li et al., 2024; Keshavarzi Arshadi et al., 2022). While dedicated anti-TB studies that combine all these elements into a single, validated pipeline are still emerging, the convergence of these well-established modules strongly supports the proposed framework as a logical evolution of computational drug discovery (Canales et al., 2024; Perveen and Sharma, 2022). Future research efforts should prioritize the establishment of standardized benchmarks to empirically quantify the incremental value delivered by each layer of this multi-scale cascade.

The diagram summarizes the integrated application of network pharmacology, ML, molecular docking, and MD simulation in anti-TB drug discovery. Key components include target identification, AMPs screening via SVM, Bayesian-based literature mining, molecular docking of repurposed drugs and novel inhibitors, and dynamic simulation of drug-target interactions. This multi-modal computational strategy accelerates anti-TB drug development across target discovery, lead optimization, and mechanism validation. TB, Tuberculosis; SVM, Support Vector Machine; AMPs, Antimicrobial Peptides; Bayesian, Bayesian algorithm; WGS, Whole Genome Sequencing; SHAP, SHapley Additive exPlanations.

## 4 Conclusion

In summary, the multi-scale computational method can develop in the following directions in the field of anti-TB research: First, generative AI and Self-driving Labs: by combining Generative Adversarial Networks (GANs) or Large Language Models (LLMs), *de novo* design of entirely new anti-TB molecules can be achieved, which is directly connected to an automated synthesis platform, significantly shortening the “design-synthesis-testing” cycle. Second, deep Computation of HDT: As mentioned above, by constructing a “multi-target and multi-pathway” network of GPCR compounds regulating ferroptosis and TB infection (Luan et al., 2025). Future computation will focus more on regulating host immune responses (rather than direct bactericidal effects) to overcome the growing drug resistance. Third, Genome-Scale Metabolic Models (GSMs): Whole-cell models integrating genomics, transcriptomics, and proteomics data will be used to simulate metabolic flux changes of Mtb, thereby guiding precise combination drug strategies. Fourth, Edge Computing and Point-of-Care Diagnosis: integrating portable Raman spectroscopy with lightweight ML models (Ogunlade et al., 2024). In the future, it is expected that point-of-care diagnosis of TB and rapid detection of drug resistance will be achieved in resource-limited areas.

In summary, the integrated application of systems pharmacology, network pharmacology, molecular docking, MD simulation, and ML has established a new multi-scale paradigm for anti-TB drug research and development, covering the “macro-mesoscopic-microscopic” scale. From using network pharmacology to analyze the synergistic mechanism of multiple targets, to leveraging ML to accelerate virtual screening, and then using MD simulation to verify binding free energy at the atomic level, these technologies complement each other and significantly accelerate the process of discovering new anti-TB drugs. Although there are still challenges in data quality, computational cost, and clinical translation, with the iteration of artificial intelligence algorithms and the upgrading of computing power facilities, multi-scale computational methods will surely play an increasingly critical role in the global initiative to end the TB epidemic.
