# Supplementary material for: Research progress on the application of multi-scale computational methods in the research and development of anti-tuberculosis infection drugs
Source: Front Pharmacol. 2026 Oct 14;17:1869128. doi: 10.3389/fphar.2026.1869128 (PMC13714510; doi:10.3389/fphar.2026.1869128)
Supplement: Supplementary file 1 [file Table1.docx]

**Table S1. Summary of Major Computational Tools, Databases, and Software Packages Cited in This Review**

| Computational Scale | Platform / Software | Core Mechanism | Key Biological Applications in TB Research | Advantages | Limitations & Challenges | Ref. |
| --- | --- | --- | --- | --- | --- | --- |
| Macro (Network) | STRING | Aggregates PPIs from experiments, text mining, and co-expression | Reconstruction of Mtb–host interactomes; identification of essential nodes (e.g., FtsZ, AKT1) | Comprehensive interaction data; regular updates | Limited to known interactions; incomplete for non-model organisms | (1, 2) |
| Macro (Network) | Cytoscape | Network visualization & topological analysis | Prioritization of hub targets via centrality metrics; pathway enrichment | Highly extensible via plugins; intuitive visualization | Topological importance ≠ biological importance | (1, 3) |
| Macro (Network) | TCMSP | Curated database linking herbs–compounds–targets–diseases | Screening of natural products; ADMET profiling of phytochemicals | Integrates pharmacokinetic parameters | Bias toward Chinese medicinal compounds | (4) |
| Meso (ML) | scikit-learn | Classical ML algorithms (RF, SVM, LR) | Resistance prediction from WGS data; permeability prediction | Mature, interpretable, low compute cost | Struggles with non-linear epistasis; feature engineering intensive | (5, 6) |
| Meso (ML) | DeepPurpose | Deep neural networks for DTI prediction | Virtual screening of ultra-large libraries; de novo molecular design | High predictive accuracy; handles complex feature spaces | Black-box model; requires large training data | (7) |
| Meso (ML) | RDKit | Cheminformatics library for descriptor calculation | Feature generation for QSAR/ML models; molecular filtering | Open-source; extensive descriptor set | Requires programming expertise | (8) |
| Micro (Docking) | AutoDock Vina | Grid-based stochastic search of ligand conformations | High-throughput screening; binding pose prediction (e.g., InhA inhibitors) | Fast, open-source, widely benchmarked | Rigid receptor assumption; scoring function inaccuracies | (9), (10) |
| Micro (Docking) | Glide (Schrödinger) | Hierarchical docking with conformational sampling | Precision docking for lead optimization; induced-fit docking | High accuracy; robust scoring | Commercial license; computationally heavier | (11) |
| Micro (Docking) | GOLD | Genetic algorithm for pose sampling | Challenging targets (metal-binding sites, flexible loops) | Excellent handling of ligand flexibility | Scoring functions still approximate | (11) |
| Micro (MD | GROMACS | Numerical integration of Newtonian equations of motion | Stability validation of docking poses; resistance mutation effects (KatG, RNA Pol) | High performance; GPU acceleration | Force field limitations; finite sampling | (12, 13) |
| Micro (MD | AMBER | All-atom MD with advanced force fields | Free energy calculations (MM/PBSA); membrane protein simulations | Accurate energetics; mature free-energy ecosystem | Steeper learning curve; high computational cost | (14, 15) |

1.	Yang Y, Fu Y, Sheng S, Ji C, Pu X, Xu G. Screening for diagnostic targets in tuberculosis and study on its pathogenic mechanism based on mRNA sequencing technology and miRNA-mRNA-pathway regulatory network. Front Immunol. 2023;14:1038647.

2.	Suresh M, Sai KV, Mitra K, Ravindran R, Doble M. A network pharmacology-based approach to understand the mechanism of action of anti-mycobacterial activity of Acacia nilotica: a modelling and experimental study. Mol Divers. 29. Netherlands2025. p. 2227–42.

3.	Luan X, Qian C, Li H, Ren W, Yao C, Shang Y, et al. Deciphering mechanisms of GPCR compounds against tuberculosis through ferroptosis inhibition using high-throughput screening and network pharmacology analysis. Bioorg Chem. 2025;163:108674.

4.	Yao L, Zhang X, Huang C, Cai Y, Wan CC. The Effect of Citrus aurantium on Non-Small-Cell Lung Cancer: A Research Based on Network and Experimental Pharmacology. Biomed Res Int. 2023;2023:6407588.

5.	Shahab M, Danial M, Duan X, Khan T, Liang C, Gao H, et al. Machine learning-based drug design for identification of thymidylate kinase inhibitors as a potential anti-Mycobacterium tuberculosis. J Biomol Struct Dyn. 2024;42(8):3874–86.

6.	Radchenko EV, Antonyan GV, Ignatov SK, Palyulin VA. Machine Learning Prediction of Mycobacterial Cell Wall Permeability of Drugs and Drug-like Compounds. Molecules. 28. Switzerland2023.

7.	Pawar A, Deka H, Battula M, Aljawdah HM, Patil PC, Chikhale R. Integrated machine learning and physics-based methods assisted de novo design of Fatty Acyl-CoA synthase inhibitors. Expert Opin Drug Discov. 2025;20(1):123–35.

8.	Alnammi M, Liu S, Ericksen SS, Ananiev GE, Voter AF, Guo S, et al. Evaluating Scalable Supervised Learning for Synthesize-on-Demand Chemical Libraries. J Chem Inf Model. 2023;63(17):5513–28.

9.	Fawibe OO, Lawal BR, Mustafa AA, Oyelakin AS, Akinyemi OF, Bankole AO, et al. Molecular docking and pharmacokinetic studies of bioactive compounds from medicinal plants as promising inhibitory agents against Mycobacterium tuberculosis enoyl-acyl carrier protein (ACP)-reductase (InhA). In Silico Pharmacol. 13. Germany2025. p. 179.

10.	Xia X, Shen J, Dai J, Arumuagam K, Rajalakshmi K, Dinakarkumar Y, et al. Design, synthesis, bioactivity evaluation, and molecular modeling of pyridin-2-yl-pyrimidine: Potential Antimicrobial and anti-tuberculosis agents. Bioorg Chem. 2025;166:109167.

11.	Patel VP, Tripathi RKP, Mandal SD. Synthesis, Biological Evaluation, Molecular Docking Studies and ADMET Prediction of Oxindole-based Hybrids for the Treatment of Tuberculosis. Curr Comput Aided Drug Des. 21. United Arab Emirates2025. p. 517–33.

12.	Cruz JN, Costa JFS, Khayat AS, Kuca K, Barros CAL, Neto A. Molecular dynamics simulation and binding free energy studies of novel leads belonging to the benzofuran class inhibitors of Mycobacterium tuberculosis Polyketide Synthase 13. J Biomol Struct Dyn. 2019;37(6):1616–27.

13.	Kashyap A, Singh PK, Satpati S, Verma H, Silakari O. Pharmacophore modeling and molecular dynamics approach to identify putative DNA Gyrase B inhibitors for resistant tuberculosis. J Cell Biochem. 2019;120(3):3149–59.

14.	Akinpelu OI, Lawal MM, Kumalo HM, Mhlongo NN. Drug repurposing: Fusidic acid as a potential inhibitor of M. tuberculosis FtsZ polymerization - Insight from DFT calculations, molecular docking and molecular dynamics simulations. Tuberculosis (Edinb). 2020;121:101920.

15.	Sabe VT, Tolufashe GF, Ibeji CU, Maseko SB, Govender T, Maguire GEM, et al. Identification of potent L,D-transpeptidase 5 inhibitors for Mycobacterium tuberculosis as potential anti-TB leads: virtual screening and molecular dynamics simulations. J Mol Model. 25. Germany2019. p. 328.
